# Low-Temperature Stability and Sensing Performance of Mid-Infrared Bloch Surface Waves on a One-Dimensional Photonic Crystal

**DOI:** 10.1021/acsami.2c07894

**Published:** 2022-09-15

**Authors:** Agostino Occhicone, Raffaella Polito, Francesco Michelotti, Michele Ortolani, Leonetta Baldassarre, Marialilia Pea, Alberto Sinibaldi, Andrea Notargiacomo, Sara Cibella, Francesco Mattioli, Pascale Roy, Jean-Blaise Brubach, Paolo Calvani, Alessandro Nucara

**Affiliations:** † Department of Basic and Applied Sciences for Engineering, Sapienza University of Rome, via A. Scarpa, 16, 00161 Roma, Italy; ‡ Department of Physics, Sapienza University of Rome, Piazzale A. Moro, 5, 00185 Roma, Italy; § CNR-IFN, Via del Fosso del Cavaliere, 100, 00133 Roma, Italy; ∥ Synchrotron SOLEIL, L’Orme des Merisiers, Saint-Aubin, Gif-sur-Yvette Cedex F-91192, France; ⊥ CNR-SPIN and Department of Physics, Sapienza University of Rome, Piazzale A. Moro, 5, 00185 Roma, Italy

**Keywords:** Bloch surface waves, mid-infrared, materials science, spectroscopy, sensing

## Abstract

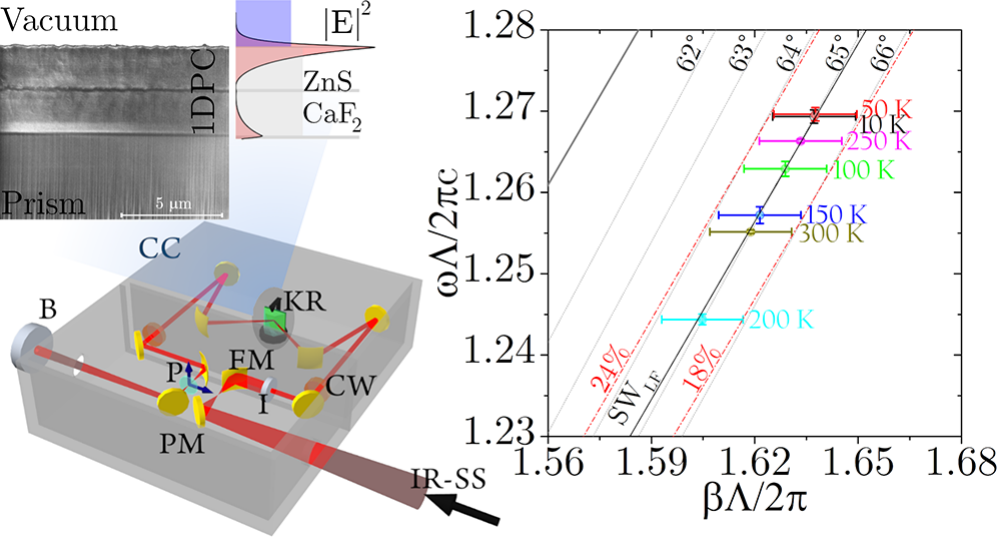

The growing need for new and reliable surface sensing methods is arousing interest in the electromagnetic excitations of ultrathin films, i.e., to generate electromagnetic field distributions that resonantly interact with the most significant quasi-particles of condensed matter. In such a context, Bloch surface waves turned out to be a valid alternative to surface plasmon polaritons to implement high-sensitivity sensors in the visible spectral range. Only in the last few years, however, has their use been extended to infrared wavelengths, which represent a powerful tool for detecting and recognizing molecular species and crystalline structures. In this work, we demonstrate, by means of high-resolution reflectivity measurements, that a one-dimensional photonic crystal can sustain Bloch surface waves in the infrared spectral range from room temperature down to 10 K. To the best of our knowledge, this is the first demonstration of infrared Bloch surface waves at cryogenic temperatures. Furthermore, by exploiting the enhancement of the surface state and the high brilliance of infrared synchrotron radiation, we demonstrate that the proposed BSW-based sensor has a sensitivity on the order of 2.9 cm^–1^ for each nanometer-thick ice layer grown on its surface below 150 K. In conclusion, we believe that Bloch surface wave-based sensors are a valid new class of surface mode-based sensors for applications in materials science.

## Introduction

Infrared (IR) spectroscopy is a powerful tool to investigate the properties of most significant quasi-particles in condensed matter by detecting their spectral “fingerprints”, i.e., their vibrational absorption lines.^1,2^ As is well known, the relevant range of wavelengths is from 2.0 to 25 μm, i.e., the mid-IR region (MIR).

In the present work, we intend to demonstrate the potential of a novel surface-enhanced IR spectroscopy scheme for application in low-dimensionality condensed matter systems at cryogenic temperatures, down to 10 K. The proposed scheme exploits the strong field localization provided by Bloch surface waves (BSWs) sustained at the interface between a finite one-dimensional photonic crystal (1D-PC) and vacuum. From this perspective, many applications can be expected for BSWs, like the extension of our precedent work on low-dimensional topological insulators^3^ and the application to the study of other features of low-dimensional systems, such as phonon overtones and combination bands in hybrid perovskites,^4^ Dirac-like dispersion of electronic states in doped graphene^5^ and spectroscopy under extreme conditions as in planetary atmospheres.^6^

In the last few decades, several strategies to improve the MIR capability to sense at the micro/nanoscale were developed and applied to a large number of practical issues, ranging from medical diagnostics to the detection of environmental pollution.^2,7,8^ Several methods make use of the strong electromagnetic field confinement occurring at the interface between different materials^9,10^ to detect molecular species in the proximity of structural boundaries.^11,12^ A large number of MIR sensors were actually fabricated, based for example on surface phonon polariton annihilation/creation in semiconductors,^13−15^ non-neutral graphene,^16,17^ and metals.^18−20^

Among such classes of novel sensors, dielectric heterostructures housing a 1D-PC have been recently investigated.^2,21^ In a 1D-PC, the refractive index modulation along the stacking direction defines an optical lattice and, accordingly, the dispersion of light propagating inside the 1D-PC^22^ is strongly distorted, as demonstrated by the appearance of a photonic band (PB) structure. PC and their photonic band (PB) have been a very intensively investigated research field.^23−25^ Less attention was paid to the excitation of BSWs at the surface of a finite PC, particularly to the electromagnetic modes localized at the boundary between a truncated 1D-PC and an external homogeneous medium.^22^ Similar to surface plasmon polaritons, BSWs are characterized by an electric field envelope, which decays exponentially both inside the 1D-PC and the external medium.^22,26,27^ BSW-based sensors working in the visible range showed enhanced resolution for the detection of molecular species in a wide range of applications.^28−30^ Surprisingly, the interest toward BSWs in the MIR region has increased only recently,^2,21^ driven by the need for overcoming the sensitivity shortcomings of MIR spectroscopy, when applied to very thin films, and on the track of previously proposed methods, such as photoexpansion^31^ and grazing-angle spectroscopy with polarized IR beams.^3,32−35^ The 1D-PC geometry offers options to design and optimize for specific applications.^36^ The PB of a 1D-PC has been demonstrated to be robust and tolerant to a significant amount of deviation from the designed structure.^37−39^ A threshold level of disorder in 1D-PCs has been found in ref (40): σ_th_ ≈ (Δω/3ω_c_)^1/2^, where σ_th_ is the width of a Gaussian distribution of the optical lengths of the PC period and Δω and ω_c_ are the photonic band gap width and photonic band gap central frequency, respectively. Below the threshold, the photonic band gap is stable in the presence of disorder.^40^ As far as we know, the robustness of surface-enhanced 1D-PC sensing schemes at extreme temperatures needed for the investigation of MIR excitations in low-dimensional systems has not been tested so far. This study is then the first mandatory step for the application of BSWs to such a field, an important test for the 1D-PC fabrication technology, and the possibility to integrate the scheme with the peculiar properties of the IR radiation emerging from the AILES beamline of the SOLEIL synchrotron.^41^

In addition to that, but not less important, this work demonstrates experimentally the BSW molecular sensing capability to detect molecular fingerprints at cryogenic temperatures, using water vapor as a molecular species, which condensates into a thin ice layer onto the 1D-PC surface even under moderately high vacuum conditions. We demonstrate that BSWs are sensitive to molecular layers of a few nanometers added on the top surface, with a phenomenological behavior that can be directly attributed to the water vapor–ice sublimation cycle.

## Materials and Methods

### 1D-PC Materials and Design

The materials of choice to fabricate the 1D-PC are CaF_2_ and ZnS; both show high transparency and a convenient refractive index mismatch in the MIR spectral range. Based on the literature values of the respective refractive indices,^42,43^ the geometry of the 1D-PC was designed and tuned to the MIR wavelength range by means of several rounds of numerical simulations (see Section S1 of the Supporting Information (SI)). The designed layer structure was CaF_2_(substrate)/[ZnS(200 nm)/CaF_2_(2300 nm)]^2^/ZnS(50 nm)/vacuum, which can sustain BSWs with a suitable dispersion, which, in agreement with the literature,^40^ is stable toward the random inhomogeneities of the thicknesses of the layers arising from fabrication uncertainty (see Section S1 and Figure S.2 of the SI).

**Figure 1:**
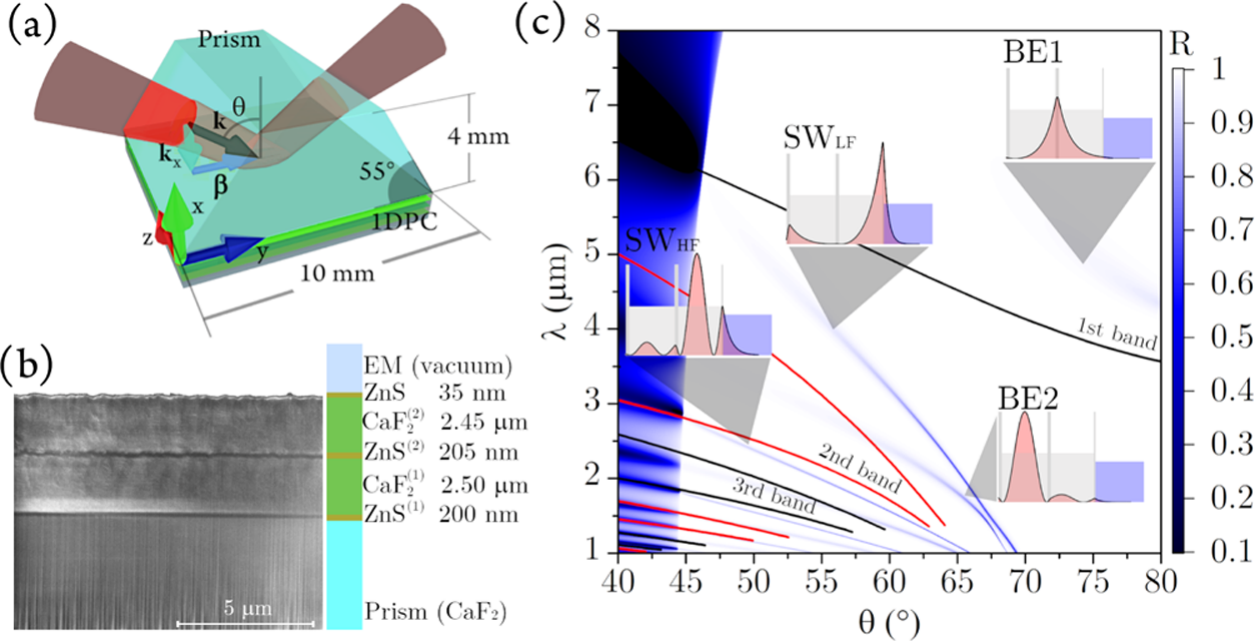
(a) Sketch of the Kretschmann–Raether configuration. The prism is quoted and the radiation wavevector ***k*** has been decomposed in its parallel, **β**, and perpendicular, ***k***_***x***_, components. (b) Scanning electron microscopy (SEM) image of the focused ion beam (FIB) milled cross-section of the deposited 1D-PC and a sketch of the transverse geometry with measured layer thicknesses. (c) *R*(θ,λ) reflectance map according to the transfer-matrix method (TMM) calculation for σ polarization. The black and red lines are the photonic band edges (BEs), calculated for the infinitely extended 1D-PC. The surface waves (SWs) and the band-edge (BE) modes appear as dark reflectance lines. In the insets, we plot the square modulus of the electric fields of the modes, superimposed on the 1D-PC refractive index distribution. The external medium is vacuum.

1D-PCs were fabricated by thermal evaporation in a high-vacuum chamber, with a limiting pressure of 5 × 10^–7^ Torr, equipped with two evaporation sources.^44^ CaF_2_ and ZnS layers could therefore be deposited without breaking the vacuum from granulated materials (UMICORE, purity 99.99%) using molybdenum boats. They were deposited on CaF_2_-truncated prisms, purchased from Korth Kristalle, with a 55° base angle and designed to operate in a Kretschmann–Raether (KR) configuration under total internal reflection (TIR) conditions^45^ (see Figure 1a). We preliminarily deposited a 20 nm thick CaF_2_ layer on the CaF_2_ substrates to improve adhesion and avoid delamination of the first ZnS layer. The main issue in the deposition of multilayers is strain accumulation that increases with the layer thickness and may cause undesirable buckling and bending.^46,47^

Preliminary test samples and the final 1D-PC were characterized by dual-beam focused ion beam (FIB) milling cross-sectional analysis, scanning electron microscopy (SEM), and near-normal incidence reflectance. SEM inspection of CaF_2_/ZnS/CaF_2_ test structures deposited on CaF_2_ substrates appeared undamaged even after immersion in liquids and after strong ultrasound treatments.^21^

The SEM image of the FIB cross-section of a 1D-PC deposited on the prism is shown in Figure 1b (for details see Section S2 in the SI). The first CaF_2_ (∼20 nm) adhesion layer is not visible in the image, while one can clearly distinguish the 1D-PC structure composed of the two ZnS/CaF_2_ binary units (dark yellow and green in the lateral sketch, respectively) and the final 35 nm thick ZnS defect layer (dark yellow). From the image, we retrieved the effective 1D-PC geometry Prism/ZnS^(1)^/CaF_2_^(1)^/ZnS^(2)^/CaF_2_^(2)^/ZnS/vacuum with measured thicknesses of 0.200(±0.015)/2.50(±0.07)/0.205(±0.025)/2.45(±0.10)/0.035(±0.020) μm, respectively. The 1D-PC surface profile, according to atomic force microscopy, had a root mean square (rms) roughness *R*_q_ of ∼ 32 nm (see Section S3 of the SI). The periodic 1D-PC defines the photonic band structure of the multilayer, whereas the additional top defect layer is used to fine-tune the BSW dispersion. Moreover, the temperature-dependency of the material refractive indices is considered through the temperature-dependent Sellmeier model reported in refs (42) and (43) for CaF_2_ and ZnS, respectively. In particular, we evaluated that the refractive indices at a wavelength of 2.1 μm (ν = 4.76 × 10^3^ cm^–1^) and at room temperature (298 K) are 2.26 for ZnS^43^ and 1.42 for CaF_2_.^42^ However, we found (see Section S4 of the SI) that the refractive indices of the ZnS and CaF_2_ layers of the 1D-PC are reduced due to the materials’ porosity. The effect of the porosity was studied by fitting the stack reflectance measured at near-normal incidence (about 8°) with unpolarized light. Making use of a Maxwell Garnett model,^48^ we evaluated the void/full ratio of the materials, which was approximately 1–2% for ZnS and 18 ± 2 and 21 ± 3% for CaF_2_^(1)^ and CaF_2_^(2)^ layers, respectively. Such porosity values correspond to a refractive index of 2.25 for the ZnS layers and 1.348 and 1.335, respectively, for the CaF_2_^(1)^ and CaF_2_^(2)^ layers.

Based on the real 1D-PC geometry and materials’ optical properties, the room temperature numerical simulation of the reflectance *R*(θ,λ) was carried out by a proprietary transfer-matrix method (TMM) MATLAB code.^26^ The numerical simulations refer to the Kretschmann–Raether configuration shown in Figure 1a, which was used experimentally to excite the BSW. As is known,^45^ BSWs can be excited only under TIR conditions for the prism/vacuum interface.^22^ In the following, **β** and ***k**_x_* are the parallel and perpendicular components of the incidence wavevector ***k***, with respect to the 1D-PC interfaces, and σ and π refer to the transverse electric and transverse magnetic polarizations, respectively.

The proposed 1D-PC sustains both σ and π BSW modes;^21^ however, in the π-polarized BSW case, the field enhancement is small due to its weak localization. We will therefore focus on the σ-polarized BSWs, both experimentally and theoretically (details of the π case are given in Section S6 of the SI). In Figure 1c, we show the calculated 1D-PC reflectance *R*(θ,λ), in the MIR wavelengths range (1 μm < λ < 8 μm) and in a wide range of incidence angles θ inside the prism, including the TIR edge (40° < θ < 80°). Moreover, in the same (θ,λ) plane, we plot, with red and black solid lines, the PB edges for σ-polarization, as calculated by means of an iterative plane wave eigensolver method.^23,49^ In such a calculation, an infinite 1D-PC was assumed, with CaF_2_ and ZnS layers’ thickness corresponding to the mean of the values found by SEM for the fabricated layers. The lines plotted with the same color are the band edges of the same *n*th order band, where the propagation inside the 1D-PC is permitted.

Beyond the TIR angle, the dispersion curves (Figure 1c) relate to different σ-polarized modes propagating along the 1D-PC structure, whose nature is identified by the insets showing the plots of the square modulus of the electric field together with the refractive-index profile *n*(*x*). From the simulation, we can see that the 1D-PC can sustain BSWs in both the first (low frequencies surface waves, SW_LF_) and in the second (high frequencies surface waves, SW_HF_) photonic band gap. In particular, the SW_LF_ modes can be excited at wavelengths lower than 6 μm and in the angular range of 47° < θ < 69°, with an intensity profile that decays exponentially from the interface with vacuum. Further, we estimate the field intensity enhancement, *f*_σ_, for the BSW, through the ratio
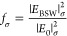
1where |*E*_BSW_|_σ_^2^ and |*E*_0_|_σ_^2^ are the maximum field intensities under TIR excitation at resonant angle θ_BSW_, for either the 1D-PC-coated prism or the bare prism, respectively. The maximum enhancement *f*_σ_ ∼ 27 is obtained at λ = 3.0 μm for θ_BSW_ = 60.4°. Moreover, the 1D-PC also admits band-edge modes which are labeled with acronyms BE1, and BE2; for such modes, the field is localized inside the 1D-PC; indeed, BE1, in the 1D-PC first permitted PB show their maximum intensity inside the ZnS^(2)^ layer, whereas the BE2 is pinned at the CaF_2_^(1)^ layer.

### Experimental Method

**Figure 2:**
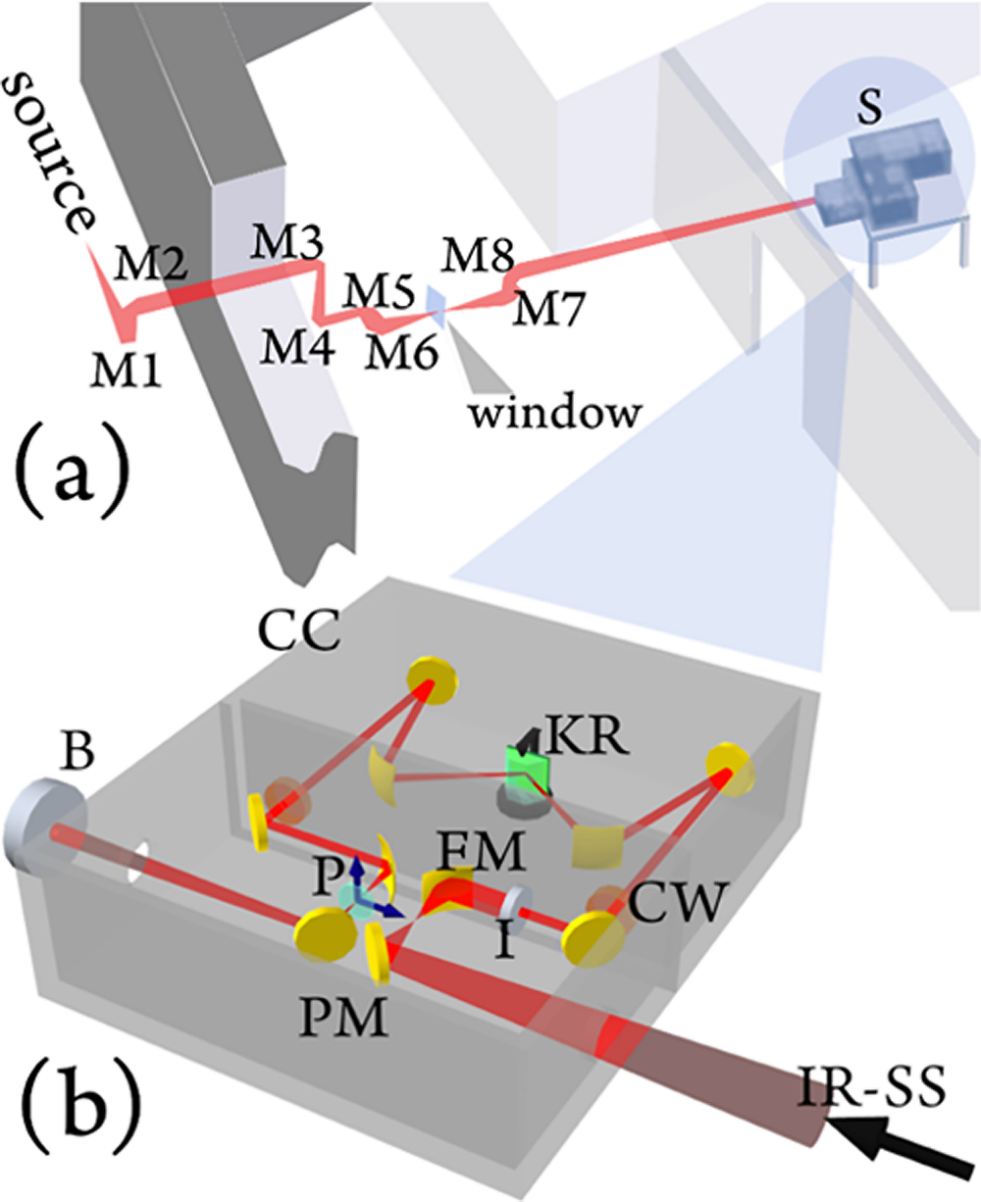
(a) Sketch of the AILES beamline at SOLEIL synchrotron which exploits a series of mirrors (M) to handle the radiation. With permission from ref (41), we reproduced a portion of Figure 4. Copyright 2006 Elsevier B.V. (b) Sketch of the experimental apparatus. Infrared synchrotron source (IR-SS), plane mirror (PM), focusing mirror (FM), iris (I), compartments window (CW), Kretschmann–Raether (KR) configuration, polarizer (P), cryoscopic compartment (CC), and bolometer (B).

In Figure 2, we sketch the apparatus used to experimentally characterize the deposited 1D-PC. We perform high-resolution reflectivity measurements using the SOLEIL synchrotron source, on the AILES beamline. A schematic sketch is shown in Figure 2a.^41^

The measurements have been performed using a Bruker IFS 125 HR interferometer equipped with a KBr beamsplitter and liquid He-cooled detectors (Figure 2a, S). In the present experiments, we used a cryoscopic chamber (CC) allowing measurements of reflectivity at variable angles, θ (grazing-angle reflectivity setup^50^), and temperatures, *T*. The prism, with or without the 1D-PC, was mounted on the closed cycle cryogenerator whose cold finger is thermally connected to the custom-made sample holder. The temperature cycles are created and controlled by a custom LabView code. Moreover, the position of the sample holder can be finely adjusted to compensate for any contraction at low temperatures.

The sample compartment of the interferometer is used here to host the optics, which redirects the radiation to the cryoscopic chamber. In Figure 2b, we sketch the grazing-incidence optical setup assembled to excite the BSW.^50^ The synchrotron IR radiation was collimated and focused by means of a series of planar (PM) and focusing (FM) gold mirrors. The spot size was reduced by means of an iris (∼1 mm, I), and the IR radiation was collected and collimated by means of a mirror system specularly mounted with respect to the input mirrors. On the path, a σ-polarizer (P) was inserted. The interferometer and the cryoscopic compartments are connected by an input and an output chamber window (CW) transparent to IR and visible radiations. The probe laser beam can therefore tunnel through the 1D-PC and efficiently excite the BSWs at the free 1D-PC/vacuum interface, as sketched in Figure 1a. The temperature in the cryostat compartment was measured by means of a thermocouple located about 2 cm above the sample, by ensuring a temperature measurement uncertainty of ±2 K.

Finally, although the ultimate vacuum in the interferometer can be reduced to 10^–4^ mbar, the sample compartment, of dimensions 262 mm × 184 mm × 162 mm, can be improved to 10^–6^ mbar, to reduce absorption due to residual gases. This pumping has been carried out by means of a set of mechanical and turbo-molecular pumps.^50^

## Results and Discussion

**Figure 3:**
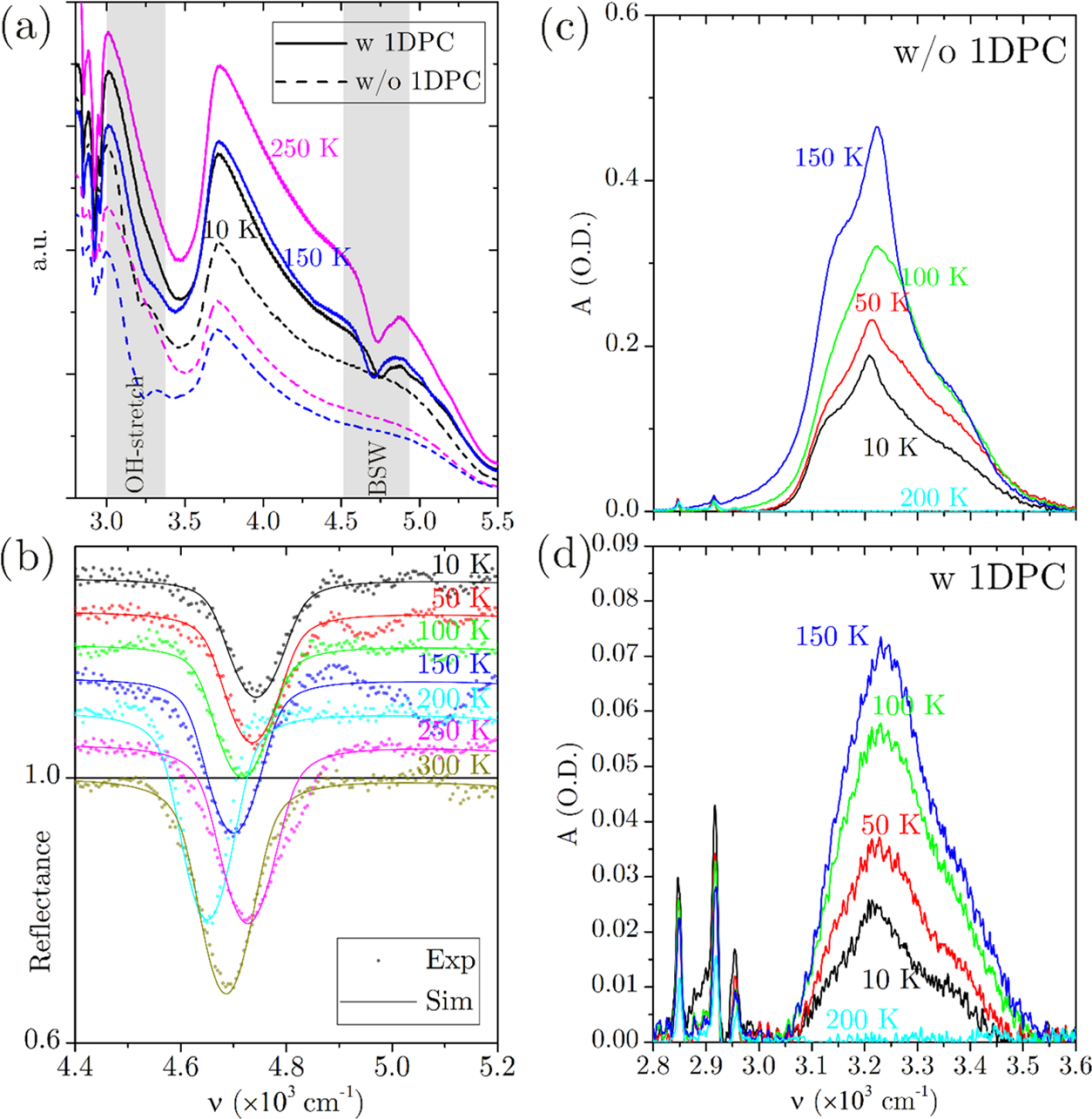
(a) Reflected intensity spectra *I*(ν,*T*) obtained at three different temperatures: (dash) bare CaF_2_ prism (reference), (solid) 1D-PC-coated CaF_2_ prism. (b) *R*(ν,*T*) reflectance spectra in the BSW excitation region: (dot) experiment, (solid) fit with the TMM simulation. Except for the case at *T* = 300 K, the curves are vertically shifted for convenience. Absorbance spectra in the OH-stretch region, for bare CaF_2_ prism (c) and of the 1D-PC-coated CaF_2_ prism (d).

The spectral intensity *I*(ν,*T*) of the σ-polarized radiation reflected by a sample was collected at the incidence angle θ_0_ = 65.0 ± 0.9° and at temperature *T*, with a spectral resolution of 0.25 cm^–1^. It is noteworthy that the incidence angle could be affected by a systematic error too, due to the difficulty in aligning the sample inside the chamber, which was estimated to be less than 1°. In Figure 3a, we show the *I*(ν,*T*) spectra for either a 1D-PC-coated CaF_2_ prism (solid lines) or a reference bare CaF_2_ prism (dashed lines), obtained at three different temperatures. For both types of samples, the following thermal cycle procedure was adopted: starting from 300 K, the temperature was gradually reduced to 100 K and suddenly increased up to 250 K, to ensure maximum evacuation of the chamber and, eventually, to forewarn for any alignment issue induced by uncompensated thermal contraction/expansion. Once completed this preliminary procedure, the temperature was lowered to 10 K and the reflectance was measured, in the order, at 10, 50, 100, 150, 200, 250, and 300 K. The experimental session has a duration of 3 h for each polarization.

In Figure 3a, we show with light gray bands two spectral regions of our interest, on which we shall focus the data analysis. In the first region, around 4.7 × 10^3^ cm^–1^, the excitation of BSWs is witnessed by the appearance of dips in the reflectance measured for the 1D-PC-coated prism. In the second region, around 3.25 × 10^3^ cm^–1^, we observed absorption dips related to the OH-stretching band.

Figure 3b shows details of the region around 4.7 × 10^3^ cm^–1^, in which the experimental reflectance curves *R*(ν,*T*) (dots) obtained at different temperatures are plotted with an arbitrary offset with respect to the 300 K measurement for the sake of clarity. The *R*(ν,*T*) curves were obtained by normalizing the experimental data to the reference curves obtained with the bare prism. The solid lines are the fits obtained with the TMM simulations. The results show that the 1D-PC continues sustaining BSWs down to 10 K without any damage to the dielectric stack, such as delamination, demonstrating their appeal for applications in extreme environmental conditions, such as space optics and spectroscopy, for example, absorption spectroscopy in planetary atmospheres.^6^ The observed frequency shift of the resonant dip with temperature indicates a perturbation of the refractive index of either the external medium or the 1D-PC itself, giving rise to a change in the BSW dispersion. We ascribe the shift primarily to the condensation of residual water vapor in the vacuum chamber to a thin ice layer at the surface of the 1D-PC at low temperatures and secondarily to the presence of residual condensed water inside the 1D-PC pores at higher temperatures. During the thermal cycle, the resonant frequency (ν_BSW_) gradually shifts as *T* increases from 10 K. However, an abrupt discontinuity is observed above 200 K since ice sublimation takes place and retrieves the real part of the refractive index on the top of the 1D-PC to that of vacuum.

The continuous growth of the thin ice film all along the experiments is confirmed by the analysis of the reflectance in the OH-stretching spectral region around 3.25 × 10^3^ cm^–1^. The increasing thickness of the water ice should not be surprising. As often happens in cryogenic measurements, albeit under high vacuum conditions, the presence of water is unavoidable. In this particular case, the main parameter which affects the ice sedimentation is the time (about 2 h) in which the sample stands below the ice sublimation temperature. In Figure 3c,d, we plot the absorbance  of either the bare or the 1D-PC-coated prism using the respective spectra acquired at 300 K as a reference assuming that the samples’ surfaces are dry at 300 K. The presence of an OH-stretching absorption peak is evident below the ice sublimation temperature at the chamber operation pressure *T*_s_ ∼ 160 K;^51^ above *T*_s_, the *A* curves are flat as confirmed by the curve at 200 K. The absorbance *A* is larger for the bare prism than the 1D-PC-coated one, approximately by a factor 7, due to the interference of the multiple reflections of the incidence wave at the multilayered structure interfaces (Bragg condition), which gives rise to a lower value of the field intensity at the 1D-PC top surface at 3.25 × 10^3^ cm^–1^ (λ = 3.08 μm), as confirmed by the TMM calculations reported in Section S5 of the SI.

From the data shown in Figure 3d, we could evaluate the thickness of the ice film. Assuming that all facets of the prism are coated with an ice film of the same thickness, using the literature values for the temperature-dependent absorption coefficient of amorphous ice at the most intense OH band,^52^ and simulating by the TMM the reflectance of the 1D-PC-coated prism, we evaluated that the thickness of the ice layer was about 5.1, 8.3, 15.5, and 21.6 nm, for the consecutive measurements taken at 10, 50, 100, and 150 K, respectively.

**Figure 4:**
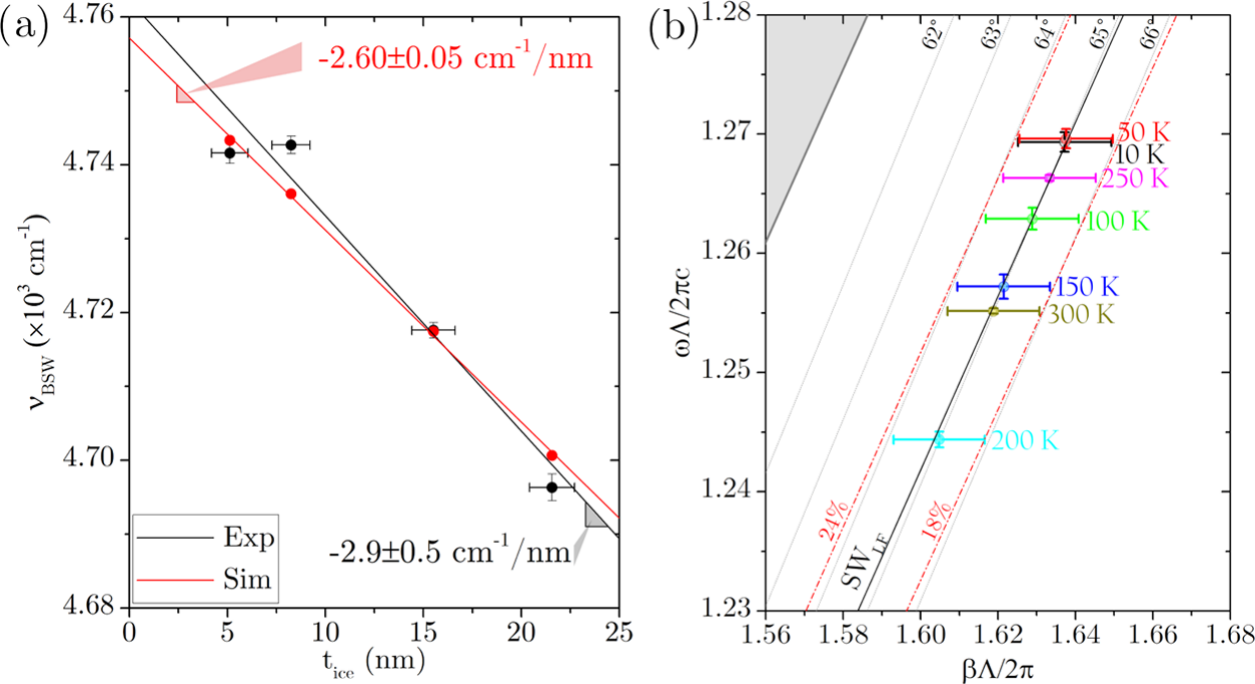
(a) Plot of the BSW peak frequency, ν_BSW_, versus the ice layer thickness for the experimental (black dots) and the simulated (red dots) cases. (b) Region of the (β̃,ω̃) plane where the experimental BSW modes are localized: (dark gray) calculated BSW dispersion (SW_LF_), (light gray) dispersion of the propagating electromagnetic waves in the prism at the fixed incidence angle θ, (dot) BSW experimental dispersion data with error bars, (red dashed) dispersion when the CaF_2_^(2)^ porosity is changed in its interval of confidence.

The values of the ice layer thickness were used to calculate the fitting curves by the TMM, as shown in Figure 3b. As for the CaF_2_ and ZnS, the ice layer has been modeled through its refractive index dispersion between 1.1 and 2.6 μm at each temperature.^52^ For completeness, we considered the possibility that the material thicknesses could change due to the thermal dilatation/contraction, but the coefficient of thermal expansion of the materials is very low for temperatures lower than the ice sublimation temperature; therefore, we considered it to be negligible. Moreover, to best fit the amplitude of the experimental curves with the simulated ones, we tuned the imaginary part of the CaF_2_ refractive index by retrieving a value almost constant in temperature in the order of 5 × 10^–5^. The agreement between the simulated curves and the experimental data until 150 K is very good and shows that the perturbation of the surface refractive index due to the thin ice layer can be efficiently detected by measuring the shift of the BSW resonance at ν_BSW_. Such a result assesses the sensing performances of the BSW mode sustained by the 1D-PC. In Figure 4a, we plot the experimental ν_BSW_ values with their uncertainty (δν_BSW_) as a function of the estimated ice film thickness *t*_ice_. Data are aligned along a straight line, whose slope is the experimental sensitivity of the BSWs toward the ice film thickness . Data are in good agreement with the TMM simulation shown as a solid line in Figure 3b, which predicts a linear dependency with a slope of −2.60 ± 0.05 cm^–1^/nm, as shown in Figure 4a. Finally, we evaluated the sensor limit of detection as the water thickness variation (Δ*t*_min_) for which the observed BSW resonance frequency variation (Δν_BSW_) is 3 times δν_BSW_, i.e., Δ*t*_min_ = 3δν_BSW_/*S*_exp_ = 1.9 ± 0.3 nm. For the sake of completeness, at ν_BSW_ = 4.76 × 10^3^ cm^–1^ (λ_BSW_ = 2.1 μm) and θ_BSW_ = θ_0_, we calculated that the field enhancement is equal to *f*_σ_ ∼ 9 and the penetration length of the electric field in a vacuum is about *l*_λ0_^σ^ = (2*k*_*x*_)^−1^ = 230 nm.

The *I*(ν, *T*) curves recorded at temperatures larger than 150 K still show a BSW resonance that, upon complete sublimation of the ice layer, shifted back to larger frequencies (measurement at *T* = 250 K). We observe extra shifts at 200 and 300 K, in opposite directions, that we cannot explain in the framework of the same model. Above the sublimation temperature, the irregular displacement of the Bloch surface wave spectral position can be due to the possible presence of water or other impurities in the voids that characterize the 1D-PC. Although at low temperatures the behavior of the water ice can be easily predicted, it is not the same for water confined into micro-nanoporosity.^53^ Therefore, we cannot formulate a hypothesis for describing what happens at temperatures higher than the ice sublimation temperature.

Finally, a detail of the 1D-PC PB structure of the nominal multilayer stack introduced above is mapped in the (β̃,ω̃) plane for σ-polarization in Figure 4b, where β̃ = βΛ/2π is the normalized parallel component of the wavevector, ω̃ = ωΛ/2πc is the normalized angular frequency, and Λ = 2.54 μm is the 1D-PC periodic unit thickness. A portion of a permitted band for light propagation is filled in gray, while the white filled area corresponds to the first photonic band gap for the electromagnetic radiation. The calculated dispersion of the BSW modes (SW_LF_) is plotted in dark gray solid line and, for the sake of clarity, we plot a grid of dotted line where each of them corresponds to a fixed incidence angle θ, i.e., the dispersion of the propagating electromagnetic waves in the prism. In the same plane, we plotted the BSW dispersion retrieved from the experiments (colored point labeled with the measurement temperature), with its experimental error bar due to the incidence angle uncertainty. The 1D-PC optical properties are very sensitive to the material porosity variation and, in particular, to the CaF_2_^(2)^ layer porosity, which is evaluated in the order of 21%, as reported above. To take into account the uncertainty of the material porosity, in the same graph, we plot the BSW mode dispersion calculated when the CaF_2_^(2)^ porosity is ranging between 18 and 24% (red dashed-dotted lines). Despite the uncertainty of the material porosity, BSWs are robust, and their observation is well reproduced by our model.

## Conclusions

In this work, we have successfully demonstrated the use of sensors based on BSWs in the mid-IR with a scheme based on the detection of both the real and the imaginary part variations of the materials’ refractive index placed on the top surface of the 1D-PC.

For the first time to our knowledge, it has been demonstrated the possibility to sustain BSWs on a 1D-PC made of CaF_2_ and ZnS layers, in the mid-IR spectral range, down to low temperatures. Although the measurements have been performed at pressures on the order of 10^–6^ mbar, we observed the formation of a water ice layer, whose thickness does not exceed 30 nm. We have exploited this effect to demonstrate that the spectral dip associated with the BSWs is able to detect the deposition of a nanometric layer on top of the 1D-PC, through variations of both the real and imaginary parts of the refractive index, which gives rise, respectively, to a shift of the spectral position and to variations of the depth/width of the dip. Ultimately, we estimated that the proposed BSW-based sensor has a sensitivity on the order of 2.9 cm^–1^ for each nanometer of icy water added to its top surface for a temperature lower than 150 K.

## Supporting Information Available

Numerical simulations, dielectric stack characterization, calculated field intensity at λ = 3.08 μm, and experimental results and numerical simulations related to the π-polarized BSWs (PDF)

## Abbreviations

1D-PC: one-dimensional photonic crystal
BSWs: Bloch surface waves
PB: photonic band
MIR: mid-infrared
σ: transverse electric polarization
π: transverse magnetic polarization
FIB: focused ion beam
SEM: scanning electron microscopy
TIR: total internal reflection
TMM: transfer-matrix method
SW_LF_: low-frequency surface waves
SW_HF_: high-frequency surface waves
BE: band-edge modes
PM: planar mirror
FM: focusing mirror
I: iris
P: polarizer
CW: chamber window
