# Supplementary material for: Low-Temperature Stability and Sensing Performance of Mid-Infrared Bloch Surface Waves on a One-Dimensional Photonic Crystal
Source: ACS Appl Mater Interfaces. 2022 Sep 15;14(38):43853–60. doi: 10.1021/acsami.2c07894 (PMC9523610; doi:10.1021/acsami.2c07894)
Supplement: Supplementary file 1 — am2c07894_si_001.pdf [file am2c07894_si_001.pdf]

Supporting Information

Low-Temperature Stability and Sensing Performance of Mid-Infrared Bloch Surface Waves on a One-Dimensional Photonic Crystal

Agostino Occhicone,[1,] Raffaella Polito,[2] Francesco Michelotti,[1] Michele Ortolani,[2] Leonetta Baldassarre,[2] Marialilia Pea,[3] Alberto Sinibaldi,[1] Andrea Notargiacomo,[3] Sara Cibella,[3] Francesco Mattioli,[3] Pascale Roy,[4] Jean-Blaise Brubach,[4] Paolo Calvani,[2] and Alessandro Nucara[5]*

[1] Department of Basic and Applied Sciences for Engineering, Sapienza University of Rome, via A. Scarpa, 16, 00161 Roma, Italy.

[2] Department of Physics, Sapienza University of Rome, Piazzale A. Moro, 5, 00185, Italy.

[3] CNR-IFN, Via del Fosso del Cavaliere, 100, 00133 Roma, Italy.

[4] Synchrotron SOLEIL, L'Orme des Merisiers Saint-Aubin, Gif-sur-Yvette, Cedex, F-91192, France.

[5] CNR-SPIN and Department of Physics, Sapienza University of Rome Piazzale A. Moro, 5, 00185, Italy.

* Corresponding author email: agostino.occhicone@uniroma1.it (orcid.org/0000-0001-8295-6222).

S1. 1DPC design

The designed layer structure, as reported in the main text, was CaF_2(substrate)/ [ZnS(200 nm)/ CaF_2(2300 nm)]× 2/ ZnS(50 nm)/ vacuum, which can sustain BSW with a suitable dispersion and enough robustness to fabrication uncertainty. Moreover, the 1DPC has been designed to ensure the largest bandwidth as possible and a deep BSW resonance dip. To evaluate the 1DPC structure properties in the Mid-IR wavelength range, we started from the material refractive index reported in literature. In particular, for the CaF_2 we used the following[1]:

$$n_{CaF_2} = \lambda\sqrt{1 + \frac{\lambda^2 a_1}{\lambda^2 - b_1} + \frac{\lambda^2 a_2}{\lambda^2 - b_2} + \frac{\lambda^2 a_3}{\lambda^2 - b_3}}$$

with:

$$a = (a_1, a_2, a_3) = (0.5675888, 0.4710914, 3.8484723)$$
$$b = (b_1, b_2, b_3) = (0.00252643, 0.01007833, 1200.555973)$$

For the ZnS, we used the following one[2]:

$$n_{ZnS} = \sqrt{g_1 + \frac{g_2}{\lambda^2 - e_1^2} + \frac{g_3}{\lambda^2 - e_2^2}}$$

with:

$$g = (g_1, g_2, g_3) = (8.393, 0.14383, 4430.99)$$
$$e = (e_1, e_2) = (0.05861241, 1347.6241)$$

For both the relations, λ is the radiation wavelength in µm.
As reported in the main text, the deposited materials are characterized by a porosity that is not neglectable. To take into account the porosity, we used the Maxwell – Garnett mixing rule[3] to calculate the refractive index of the composite material:

$$n_{MG}^2 = \varepsilon_{MG} = \varepsilon_0 \frac{2(1 - v_i)\varepsilon_0 + (1 + 2v_i)\varepsilon_i}{(2 + v_i)\varepsilon_0 + (1 - v_i)\varepsilon_i}$$

where v_i is the fraction volume of the inclusions, ε_i the dielectric constant of the included material and ε_0 the dielectric constant of the matrix material. For numerical simulations, we considered a void fraction volume of 25% and 0% for CaF_2 and ZnS, respectively.
As well-known, a multilayer stack characterized by a periodical refractive index function shows the opening of photonic band gaps where the light cannot propagate, as extensively discussed in literature[4]. In Figure S.1(a), we reported the reflectance map carried out by means of the numerical simulation by exploiting the TMM for the structure mentioned above. As shown in figure, when in total internal reflection, localized modes become possible, in particular, the proposed 1DPC is characterized by a BSW dispersion which span from $1.5\ \mu m$ to $5.5\ \mu m$ in wavelength and from $47°$ to $65°$ in angle. By choosing $\lambda_0 = 2.5$ µm, and $\vartheta = 60$ deg, we observe the deepest BSW resonance dip, i.e. the reflected light is not larger than the 5% (as shown in Figure S.1(b)).
In Figure S.1(b), we reported the reflectance profile for the structure for fixed wavelength in some exemplary cases. Here, it is tracked the BSW position by an arrow and it can be observed that the BSW resonance dip changes its shape and angular position when increasing the wavelength.

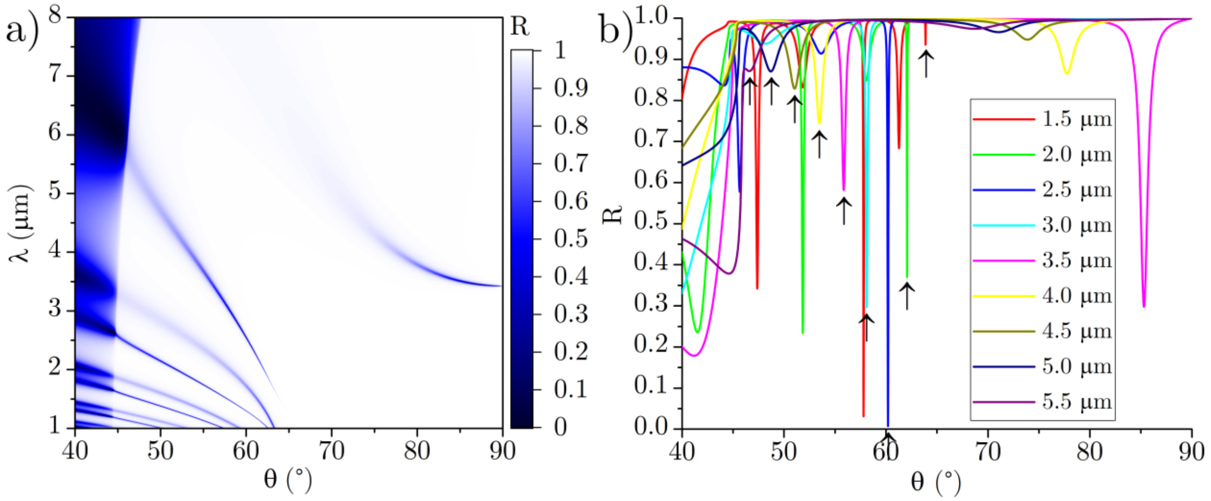

Figure S.1. a) Reflectance map of the designed 1DPC. b) The reflectance profile calculated for some wavelenghts. The arrow highlight the BSW resonance dip in the first photonic band gap.

For sake of completeness, in Figure S.2, we plot the main features of the BSW excited on the top surface of the 1DPC when changing the materials thicknesses. We calculated the confinement (a) and the propagation (b) figure of merit (FoM) of the 1DPC as reported in ref. (5):

$$\mathrm{FOM}_{prop} = \frac{l_m}{\lambda_{BSW}}$$

$$\mathrm{FOM}_{conf} = \frac{\lambda_0}{l_d}$$

The BSW propagation distance (l_m), is obtained by using the following:

$$l_m = \frac{\lambda}{2\pi n_p \Delta\theta \cos\theta_{BSW}}$$

where θ_{BSW} and $\Delta\theta$ are the BSW resonance angle and the reflectance dip full-width half maximum (FWHM) and n_p is the prism refractive index. On the contrary, the surface electromagnetic penetration depth (l_d) is carried out by using the following one:

$$l_d = \frac{1}{2k_\perp} = \frac{\lambda}{4\pi\sqrt{n_p^2 \sin^2\theta_{BSW} - 1}}$$

where $k_\perp$ is the wavevector component perpendicular to the multilayer interfaces.

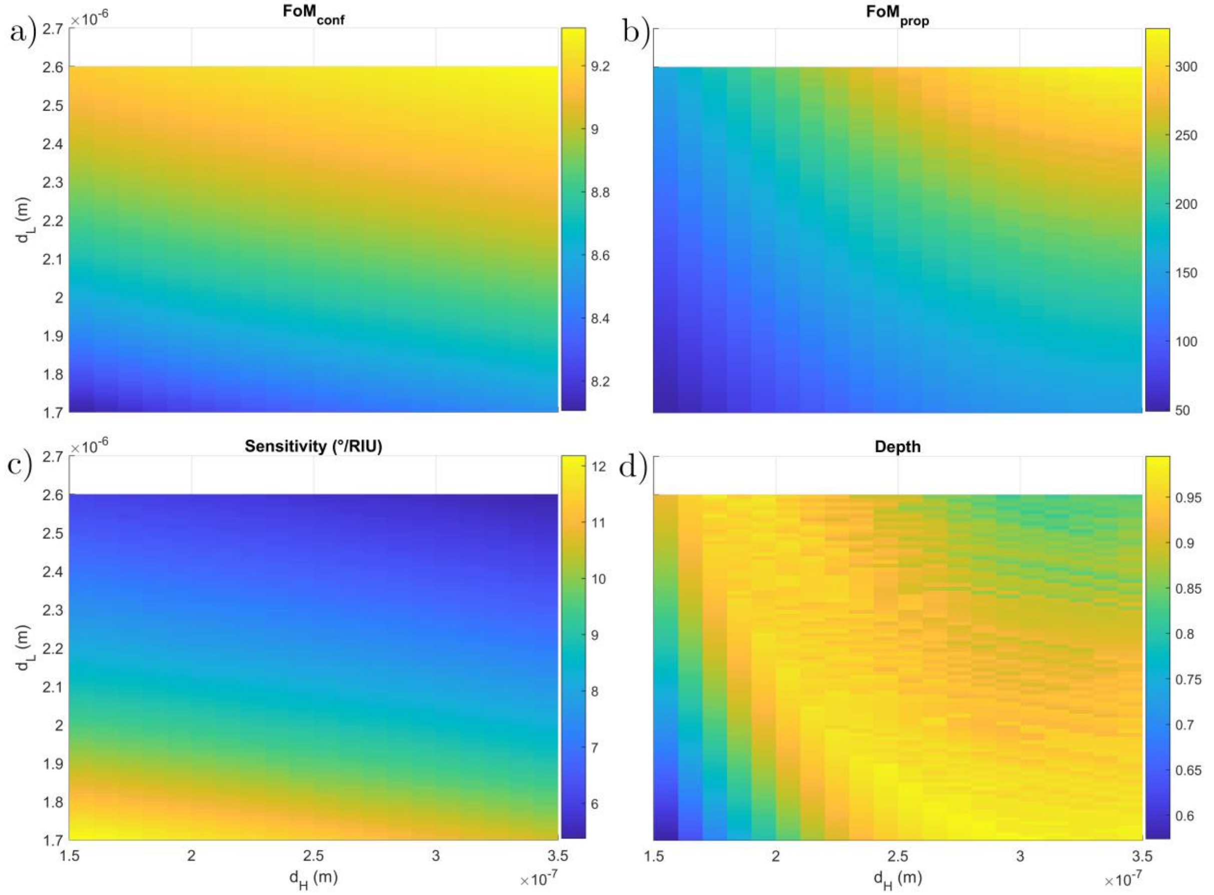

Figure S.2. Plot of the main characteristics of the 1DPC calculating when changing the materials thicknesses. We plot: (a) confinement FoM, (b) propagation FoM, (c) sensitivity and (d) the resonance dip's depth.

Moreover, we reported the structure sensitivity (in °/RIU) (c) and the depth (d) of the reflectance dip when the BSW is excited. The first observation that one can do from the calculations is the fact that the 1DPC shows a BSW also when a large change in the material thicknesses is applied to the nominal structure, i.e. the structure is robust to any change due to the fabrication uncertainty. In conclusion, the proposed structure shows the following characteristics:

$$FoM_{conf} = 9.0$$
$$FoM_{prop} = 179$$
$$S = 7.32\ °/RIU$$
$$Depth = 0.94$$

S2. SEM image of the FIB cross-section

In each multilayer deposition run, an additional CaF_2 flat substrate (½-inch-diameter window) was placed close to the CaF_2 prism substrate used for IR measurements. The FIB/SEM investigation showed that the multilayer samples deposited on both substrates in the same deposition run have the same characteristics and thickness on the entire surface apart from few microns from the borders. Therefore, the accurate analysis of the FIB/SEM sections has been carried out from multi-layered structure deposited on the respective CaF_2 flat substrate, thus keeping unaltered the prism substrate to be used for IR measurements. In any case, after the IR characterization, the multilayer deposited on the CaF_2 prism has been characterized by means of FIB/SEM measurements involving an area not larger than 400 μm^2 far from both the IR measurements area and the prism edge. The shallow ion-beam-induced damaged layer is localized to this area and, given the Ga^+ ion energy of 30keV, has a thickness of few tenths of nanometer.

S3. Dielectric stack: AFM characterization

The deposited multi-layered structures have been characterized by atomic force microscopy (AFM) as already discussed in our previous work (ref. (5)) and which we report here for clarity. Films with different thickness have been deposited to follow the morphology evolution through the whole deposition process. In particular, CaF_2 layers with increasing thickness (110, 1850 and 2750 nm respectively) evaporated on CaF_2 substrates show clearly a columnar triangular arrangement. On the other hand, the ZnS layers deposited on the CaF_2 substrates, with the CaF_2 adhesion layer, maintain a flat and homogeneous surface even for large thickness. In Figure S.3, we report the surface morphology of the multilayer described in the main text (with structure from top to bottom ZnS/ CaF_2/ ZnS/ CaF_2/ ZnS/ CaF_2(adlayer)/ CaF_2 (substrate)) and carried out by means of AFM measurements on an area of $1 \times 1\ \mu m$. Apparently, the triangular columnar structures, distinctive of the CaF_2 layers, are still quite well visible[5].
The surface morphology evolution can be quantitatively described in terms of the RMS surface roughness analysis, as shown in the graph of Figure S.4 [5]. It is apparent that upon increasing the thickness, the roughness of the deposited CaF_2 (black square dots) increases up to a value of about 30 nm. Conversely, the roughness of the deposited ZnS layers (red circle dots) decreases from an initial value of about 3 nm to values between 1-2 nm. In the same graph, we placed the roughness measured for the multi-layered structure, which is evaluated in the order of 32 nm, as mentioned in the main text.

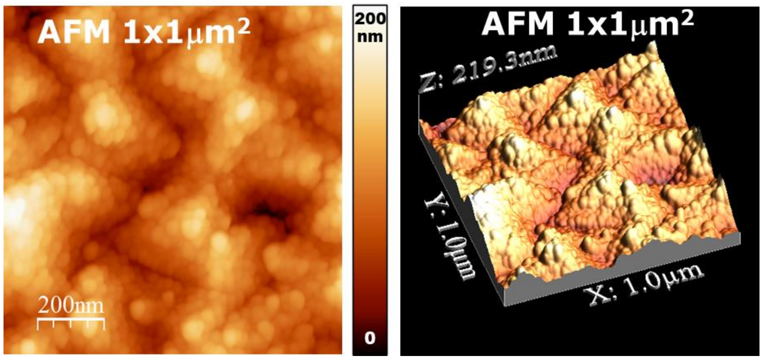

Figure S.3. AFM images (1×1 μm) of the deposited multilayer: an intensity plot on the left and a 3D rendering of the surface on the right. Reproduced from ref. (5). Copyright © 2020 American Chemical Society.

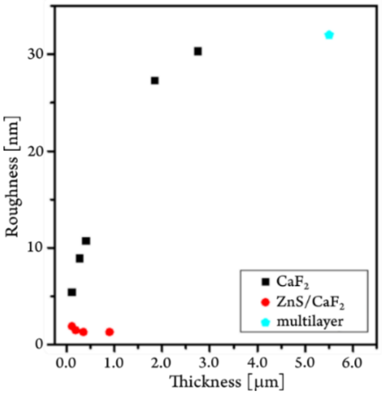

Figure S.4. Surface roughness of deposited CaF_2 (black square dots), ZnS (red circle dots) and of the multilayered structure (pentagonal light blue dot). In the same graph, the roughness of the multi-layered structure, described in the main text, is reported (pentagonal light blue dot). This roughness value is compatible with the saturating behaviour of the CaF_2 roughness. Reproduced from ref. (5). Copyright © 2020 American Chemical Society.

S4. Dielectric stack characterization

The materials' stack refractive index values have been retrieved by fitting the data obtained by measuring the stack reflectance in the near-normal incidence of an unpolarized radiation beam (about 8° far from the normal incidence) through a custom MATLAB code based on a Fresnel multilayer model (Figure S.5). The latter one assumes the multilayer constituted by homogeneous materials with perfectly flat interface and with a refractive index that is evaluated by using the Maxwell-Garnett mixing rule, where the refractive index dispersion of the pure material has been obtained from literature[6,7].

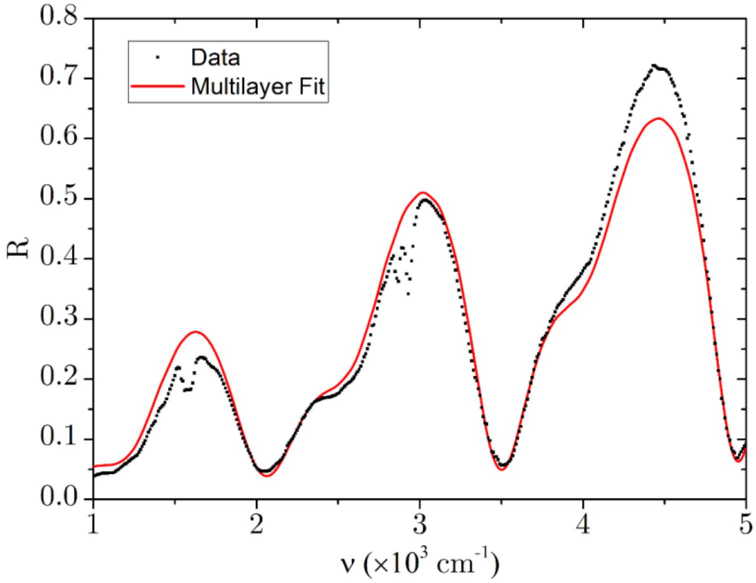

Figure S.5. Reflectance measurements performed with unpolarized IR radiation at near-normal incidence (8°±7° incidence angle) on the multilayer compared with the fitting curve obtained with the curve carried out by means of the Fresnel multilayer model.

S5. Calculated field intensity at $\boldsymbol{\lambda}$=3.08 $\boldsymbol{\mu}$m

In Figure S.6, we report the square modulus of the electric fields carried out by means of TMM associated with the total internal reflected electromagnetic wave (λ=3.08 μm) at the prism top surface with (w 1D-PC, solid line) and without (w/o 1D-PC, dotted line) the 1D-PC.

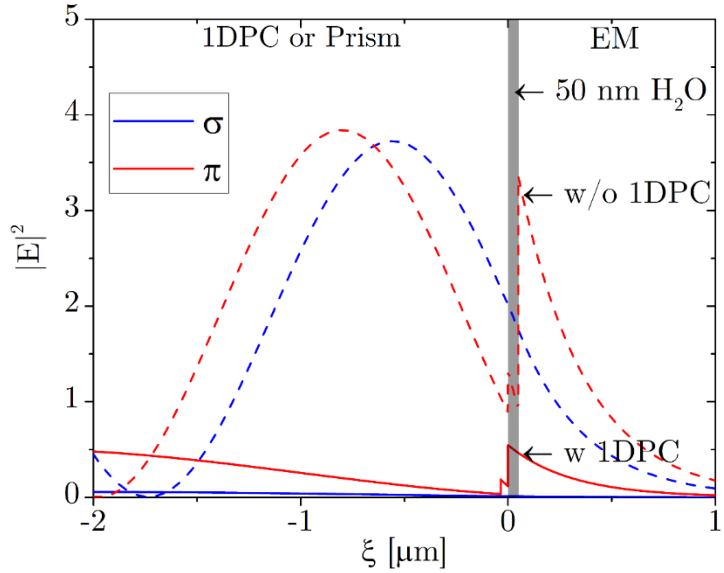

Figure S.6. Calculated square modulus of the electric fields which characterize the total internal reflected electromagnetic wave at the prism top surface with (w 1D-PC, solid line) and without (w/o 1D-PC, dotted line) the 1D-PC. The grey area highlights the water ice layer; for $\xi \leq 0$, it is plotted the square modulus of the electric field inside the 1D-PC/prism, and for $\xi > 0$, it is plotted the square modulus of the electric field inside the EM.

The grey area highlights the 50 nm thick water ice layer. For $\xi \leq 0$, it is plotted the square modulus of the electric field inside the 1D-PC or inside the prism, depending on the case, and for $\xi > 0$, it is plotted the field inside the external medium. The electric field has been evaluated for both the σ (black curves) and the π (red curves) polarization.
By integrating the field intensity in the range of the ice water layer ($\boldsymbol{\Sigma}$), we can quantify the interaction strength of the electromagnetic wave with the ice water layer. We obtained the following:

	w 1D-PC $\boldsymbol{\Sigma_w}$ $[\boldsymbol{a.u.\cdot m}]$	w/o 1D-PC $\boldsymbol{\Sigma_{w/o}}$ $[\boldsymbol{a.u.\cdot m}]$	$\frac{\boldsymbol{\Sigma_w}}{\boldsymbol{\Sigma_{w,o}}}$
σ	4.7×10^{-10}	9.4×10^{-8}	0.005
π	2.5×10^{-8}	5.6×10^{-8}	0.45

We concluded that the intensity field inside the ice water layer is about 200 times stronger when the prism is not topped by the 1D-PC in σ polarization and 2 times stronger in π polarization, by explaining the larger water ice absorption in Figure 2(c) with respect to (d).

S6. π-polarization: experimental and numerical simulation results

For sake of completeness, in Figure S.7, we plot the reflectance map simulated (a) and the experimental reflectance obtained in the π polarization case in the range $(4\text{-}6)\times10^3$ cm^{-1} (b). The map in (a) has been calculated by means of the TMM using the materials refractive index dispersion provided by literature at room temperature[6,7]. Into the map, we highlighted the propagating mode which characterize the 1D-PC when operating with a source π-polarized. The reflectance reported in (b) have been performed immediately after the σ-polarized measurements and without breaking the sample chamber vacuum.

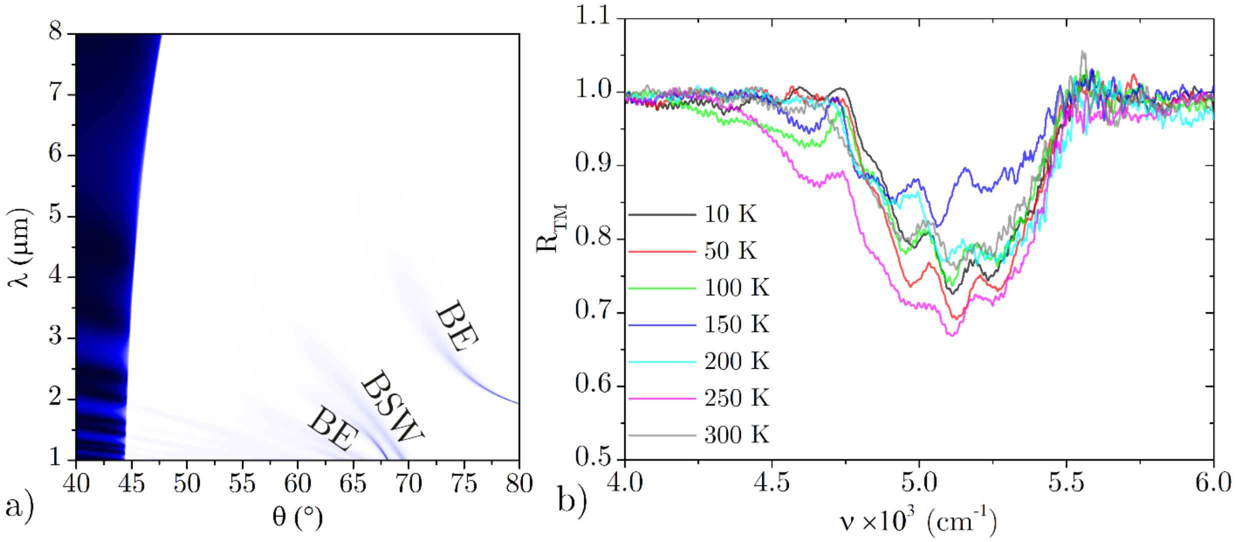

Figure S.7. a) Calculated reflectance map obtained for the π-polarized radiation. The color map ranges from 0 (black) to 1 (white). b) Plot of the reflectance profiles obtained for different temperatures as wavelength function for the π-polarized radiation.

Around $\nu=5.1\times10^3$ cm^{-1}, where BSW modes are expected (as shown in Figure S.7), there is not a clear peak that can be associated to the excitation of the BSW mode. Here, we observed a larger peak superimposed to a series of less intense peaks that we could associate to the O-H vibrational overtone spectrum of the water dimer ranging between 5.1×10^3 and 5.3×10^3 cm^{-1}, which generally show a very low intensity[8]. The anomalous intensity of this overtone suggests the presence of other contributions, likely due to the 1D-PC modes.

ABBREVIATIONS

1D-PC, one-dimensional photonic crystal; BSW, Bloch surface waves; MIR, Mid-infrared; σ, transverse electric polarization; π, transverse magnetic polarization; TMM, transfer-matrix method.

REFERENCES

[1] Malitson, I.H., "A Redetermination of Some Optical Properties of Calcium Fluoride," *Appl. Opt.,* 1963, 2, 1103-1107.

[2] Debenham, M., "Refractive Indices of Zinc Sulfide in the 0.405-13-µm Wavelength Range," *Appl. Opt.,* 1984, 23, 2238-2239.

[3] Cohen, R.W., Cody, G.D., Coutts, M.D., and Abeles, B., "Optical Properties of Granular Silver and Gold Films," *Phys. Rev. B,* 1973, 8, 3689.

[4] Joannopoulos, J., Johnson, S.G., Winn, J.N., and Meade, R.D., "Photonic Crystals," Princeton: Princeton University Press, 2008.

[5] Occhicone, A., Pea, M., Polito, R., Giliberti, V., Sinibaldi, A., Mattioli, F., Cibella, S., Notargiacomo, A., Nucara, A., Biagioni, P., Michelotti, F., Ortolani, M., and Baldassarre, L., "Spectral Characterization of Mid-Infrared Bloch Surface Waves Excited on a Truncated 1D Photonic Crystal," *ACS Photonics,* 2021, 8, 350–359.

[6] Leviton, D.B., Frey, B.J., and Madison, T.J., "Temperature-Dependent Refractive Index of CaF2 and Infrasil 301," *Proceedings Cryogenic Optical Systems and Instruments XII,* vol. 669204, 2007.

[7] Li, H.H., "Refractive Index of ZnS, ZnSe, and ZnTe and Its Wavelength and Temperature Derivatives," *J. Phys. Chem.,* 1984, 13.

[8] Salmi, T., Hanninen, V., Garden, A.L., Kjaergaard, H.G., Tennyson, J., and Halonen, L., "Calculation of the O-H Stretching Vibrational Overtone Spectrum of the Water Dimer," *J. Phys. Chem. A,* 2008, 112, 6305–6312.
